# Bio-interventional Uveoscleral Outflow Enhancement Surgery for Primary Open-Angle Glaucoma: 2-Year Results of Cyclodialysis with Scleral Allograft Reinforcement

**DOI:** 10.1016/j.xops.2025.100727

**Published:** 2025-02-03

**Authors:** Ernesto Calvo, Ticiana De Francesco, Lautaro Vera, Farrell Tyson, Robert N. Weinreb

**Affiliations:** 1 Panama Eye Center, Panama City, Panama; 2 John A. Moran Eye Center, University of Utah, Salt Lake City; 3 Clinica de Olhos De Francesco, Fortaleza, Brazil; 4 Hospital de Olhos Leiria de Andrade, Fortaleza, Brazil; 5 Tyson Eye, Cape Coral, Florida; 6 Shileye Eye Institute, University of California San Diego, San Diego

**Keywords:** Cyclodialysis, Open-angle glaucoma, Scleral allograft

## Abstract

**Objective:**

To evaluate safety and efficacy of the scleral allograft–reinforced cyclodialysis through 24 months of follow-up.

**Design:**

Interventional single-center case series.

**Participants:**

Thirty-one eyes with primary open-angle glaucoma and visually significant cataracts underwent bio-interventional cyclodialysis surgery with scleral allograft reinforcement combined with phacoemulsification.

**Intervention:**

Uveoscleral outflow enhancement surgery comprised of cyclodialysis with sequential bio-reinforcement with a scleral allograft combined with phacoemulsification.

**Main Outcome Measures:**

The primary outcome was the proportion of eyes achieving ≥20% intraocular pressure (IOP) reduction with same or fewer medications compared with baseline. Secondary outcomes included the mean change in medicated IOP and mean number of IOP-lowering medications compared with baseline. Adverse events were also recorded and evaluated throughout the study.

**Results:**

The primary outcome was achieved in 74% of the eyes, and there was a mean IOP reduction of 34% compared with baseline. Baseline mean medicated IOP was 21.9 ± 4.92 mmHg on 1.22 ± 1.29 IOP-lowering medications. At 12 months postoperation, mean IOP was 12.62 ± 2.63 on 0.55 ± 0.52 glaucoma medications. The procedure was well tolerated, and there were no serious ocular adverse events.

**Conclusions:**

Uveoscleral outflow enhancement can be successfully achieved at the time of cataract surgery through bio-interventional cyclodialysis and scleral allograft reinforcement to lower IOP in patients with primary open-angle glaucoma.

**Financial Disclosure(s):**

The author(s) have no proprietary or commercial interest in any materials discussed in this article.

Intraocular pressure (IOP) is a key risk factor for glaucoma progression, and reducing IOP remains the cornerstone of glaucoma therapy.^1^ Surgical treatments for glaucoma primarily focus on reducing the IOP by enhancing aqueous humor outflow. Unlike traditional incisional surgeries, such as trabeculectomy and glaucoma shunt implants, the current surgical paradigm has shifted toward safer minimally invasive interventional ab-interno techniques.^2^, ^3^, ^4^ These approaches now represent the majority of glaucoma surgeries performed in the United States.^5^

Most modern ab-interno surgical techniques target only 1 of the 2 major outflow pathways of the eye—the trabecular outflow pathway—while the uveoscleral outflow pathway still lacks commercially available, United States Food and Drug Administration–approved surgical treatments.^6^^,^^7^ This stands in stark contrast to the pharmacotherapy approach, where prostaglandin analogs, the first-line and most effective class of topical glaucoma therapy, act primarily through the uveoscleral pathway.^8^ There is a significant need for more efficacious glaucoma interventions, as most existing trabecular outflow procedures such as goniotomy, canaloplasty, and trabecular microstents offer only modest efficacy.^9^, ^10^, ^11^ The larger outflow capacity of the uveoscleral pathway may address the need for higher efficacy, particularly in moderate to advanced cases.^12^

Cyclodialysis, first introduced by Dr Max Heine in 1905, was the first clinically meaningful surgical intervention targeting the uveoscleral outflow pathway.^12^, ^13^, ^14^ While its effect on outflow and IOP is well documented, its long-term efficacy and durability remain limited, particularly in those eyes with premature closure of the supraciliary filtration conduit.^12^^,^^15^ Attempts to maintain the patency of the cyclodialysis over time, such as injecting stabilization materials like viscoelastic or air, have had limited success due to their transient effect.^16^

More recently, advancements address the need for a durable biocompatible reinforcement of the cyclodialysis cleft.^17^^,^^18^ One promising approach is cyclodialysis reinforced with an allograft bio-scaffold that is designed to ensure long-term stability of the cyclodialysis conduit, enhancing uveoscleral outflow and reducing IOP.^17^^,^^19^ This procedure combines cyclodialysis with highly targeted submillimeter intraocular scleral reinforcement to sustain the cyclodialysis conduit for prolonged uveoscleral outflow augmentation and IOP reduction.^17^^,^^19^

Scleral grafts have long been used for homologous scleral reinforcement in glaucoma surgery, particularly to cover the tubes of glaucoma drainage devices.^20^ The sterile acellular donor tissue is widely available, is highly biocompatible, and offers long-term durability and structural stability—key properties for an implantable bio-scaffold.^21^^,^^22^ Allogeneic scleral tissue is also hydrophilic, porous, and inert, with biomechanical properties closely resembling those of native sclera.^23^, ^24^, ^25^ This similarity reduces fibrosis and foreign body reactions, which are often driven by the stiffness mismatch between implant materials and recipient tissues.^21^^,^^23^ Unlike traditional inorganic implants, natural scleral allografts are bio-conforming and homologous to the recipient's endoscleral wall, potentially reducing fibrosis and cleft closure.^26^

This study represents a clinical investigation and report of the 2-year efficacy and safety of bio-interventional cyclodialysis surgery using allogeneic scleral reinforcement in patients with open-angle glaucoma.

## Methods

This is a single-center prospective case series of 31 consecutive surgeries of bio-reinforced cyclodialysis combined with phacoemulsification. Safety and efficacy were evaluated through 24 months of follow-up. The study protocol adhered to the tenets of the Declaration of Helsinki and was approved by the Panama Ministry of Health and the local hospital institutional review board. All study participants provided written informed consent before initiating study procedures.

### Eligibility Criteria

Inclusion criteria for the study included a confirmed diagnosis of primary open-angle glaucoma with Schaeffer grade >2 angles, patients aged >18 years old, and visually significant cataract. Primary open-angle glaucoma diagnosis was established by the presence of both glaucomatous optic neuropathy and perimetric defect consistent with open-angle glaucoma. After meeting the inclusion and exclusion criteria, patients underwent a cyclodialysis intervention with the CycloPen microinterventional system followed by scleral reinforcement with the AlloFlo allogeneic bio-scaffold (Iantrek, Inc) in combination with cataract surgery. Patients were then followed prospectively for 24 months. Exclusion criteria were the presence of narrow-angle glaucoma, prior laser or surgical iridotomy, axial length >26.0 mm, active ocular inflammation, previous incisional glaucoma surgery, clinically significant corneal opacity, or visual field loss within central 10°.

### Bio-reinforced Cyclodialysis Technique and Instrumentation

**Figure 1:**
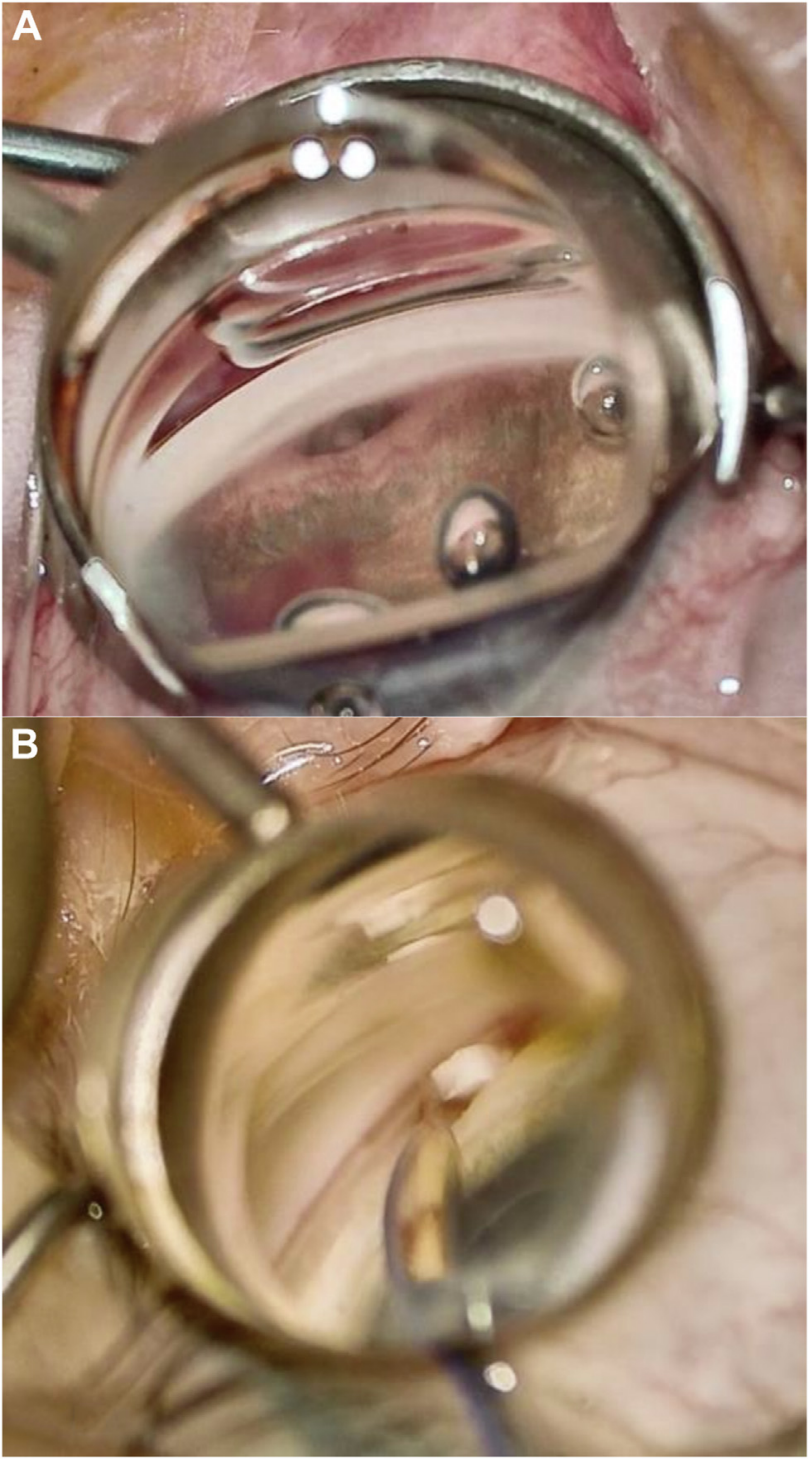
Cyclodialysis creation and viscocycloplasty augmentation (**A**). Bio-interventional allograft scleral reinforcement (**B**).

Phacoemulsification was performed first, followed by intraocular lens implantation and then the glaucoma procedure as described here. The glaucoma surgical procedure involved a 2-step sequential intervention performed under standard gonioscopic visualization and a viscoelastic-filled anterior chamber. The first surgical step was the creation of an ab-interno cyclodialysis (Fig 1A) of ≥1 clock hour to create the desired ab-interno aqueous filtration reservoir using a cyclodialysis spatula. This was followed by the injection of a cohesive viscoelastic in the cleft for additional viscocycloplasty to expand the internal uveoscleral filtration channel. The second bio-interventional step involved targeted endoscleral reinforcement, where the allograft bio-scaffold was deployed at the endoscleral surface above the ciliary body using the CycloPen system (Fig 1B). The ab-interno scleral reinforcement extended 5 mm posteriorly to maintain the cleft's entire depth and enhance the structural stability of the uveoscleral outflow channel. Proper deployment of the bio-tissue within the cyclodialysis cleft was confirmed gonioscopically, ensuring it was flush with the iris root. Viscoelastic was removed from the eye, and the eye was pressurized with a balanced salt solution. Standard-of-care postoperative treatment for cataract surgery was prescribed in all cases with topical antibiotic and anti-inflammatory agents for 4 weeks after surgery.

### Allograft Material Preparation

The allograft material was prepared from standard sterile donor scleral patch grafts obtained from an eye bank. The tissue was microtrephined using the high-precision AlloFine trephination instrumentation (Iantrek, Inc) to create a microscaffold measuring 500 μm in width and 5 mm in length for endoscleral reinforcement. This bio-scaffold was then loaded into a cannulated carrier, which was attached to a cyclodialysis system (CycloPen, Iantrek, Inc) for ab-interno deployment.

### Main Outcomes

The primary outcome was the proportion of eyes achieving ≥20% IOP reduction with same or fewer medications compared with baseline. Secondary outcomes included the mean change in medicated IOP and the mean number of IOP-lowering medications compared with baseline. Adverse events were also recorded and evaluated throughout the study.

### Statistical Analysis

Analytical methods for the results included descriptive statistics at the different time points expressed as means ± standard deviations, with differences versus baseline through 24 months of follow-up. Intraocular pressure and medication outcomes are analyzed using paired 2-tailed *t* tests. Categorical data are presented as numbers and percentages and were compared where indicated using Fisher exact test with 2 × 2 contingency tables. Logarithm of the minimum angle of resolution conversions were made for all Snellen visual acuity measures. Statistical significance was defined as *P* < 0.05. Statistical and graphing software included Excel (Microsoft Corp) and Prism, v.9.0 (GraphPad Software).

## Results

This study identified 31 eyes that underwent cyclodialysis intervention with the AlloFlo scleral allograft combined with cataract surgery. All cases had successful phacoemulsification with planned intraocular lens implantation followed by a focal 1 to 2 clock hours of cyclodialysis intervention and adjunct allograft scleral reinforcement—all steps done in a minimally invasive, clear-cornea, ab-interno surgical technique. The baseline characteristics of all included eyes are summarized in Table 1.

**Table 1:** Baseline Characteristics

Characteristics	Total
Sample Size, Eyes, N	31
Age, mean ± SD, yrs	70.5 ± 9.4
Sex: female, %	47%
Combined with cataract surgery, %	100%
Baseline BCVA, mean ± SD	0.7 ± 0.43
Baseline IOP, mmHg, mean ± SD	21.9 ± 4.9
Baseline IOP range, mmHg	13–31
Number of IOP-lowering drugs, mean ± SD	1.42 ± 1.29

BCV = best-corrected visual acuity; IOP = intraocular pressure; SD = standard deviation.

**Figure 2:**
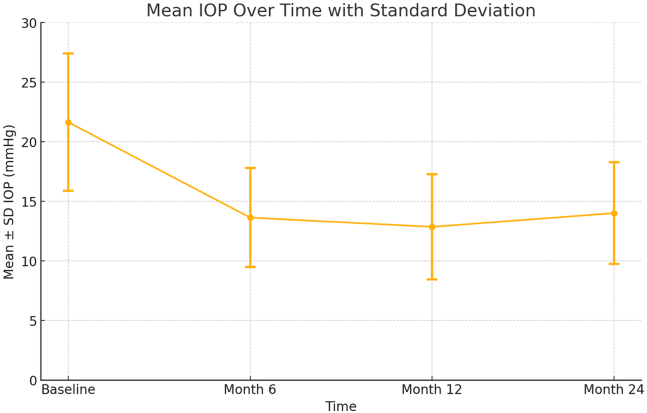
Mean medicated IOP at each time point. IOP = intraocular pressure; SD = standard deviation.

At 2 years, there was a mean IOP reduction of 34% compared with baseline and 74% of the eyes achieved success with an IOP reduction of ≥20% using the same or fewer medications compared with baseline. In addition, 96.8% and 80.6% subjects achieved 24-month IOP ≤18 mmHg and IOP ≤15 mmHg, respectively. Mean IOP was reduced from 21.9 ± 4.9 mmHg to 13.8 ± 2.4 mmHg (*P* < 0.0001), and the number of medications declined from 1.4 ± 1.3 to 0.5 ± 0.5 (*P* = 0.0006) (Table 2). Mean IOP at 6, 12, and 24 months is reported in Figure 2, and the changes in IOP at all follow-up time points are statistically significant compared with the baseline. Operated eyes had good visual acuity outcomes, increasing from an average baseline logarithm of the minimum angle of resolution best-corrected visual acuity of 0.70 ± 0.43 to 0.11 ± 0.11 at 24 months (*P* < 0.0001; n = 31 paired determinations), which is anticipated with the concurrent cataract surgery. Endothelial cell density (ECD) was collected on all 31 subjects at baseline (mean ECD = 2420.9 ± 334), 12 months (mean ECD = 2277.6 ± 322.2), and 24 months (mean ECD = 2196.2 ± 321.4). The mean endothelial cell loss at 12 and 24 months did not exceed 10% and was consistent with expected loss seen after cataract and microinvasive glaucoma surgery (MIGS) procedures. No eyes had significant (>30%) endothelial cell loss.

**Table 2:** Mean Medicated IOP and IOP-Lowering Medications at Each Time Point

Variable	Baseline	6 Months	12 Months	24 Months
Mean medicated IOP, mmHg + SD	21.9 ± 4.9	13.7 ± 3.4	12.6 ± 2.6	13.8 ± 2.4
IOP-lowering medications, n, mean ± SD	1.4 ± 1.3	0.6 ± 0.6	0.5 ± 0.5	0.5 ± 0.5

IOP = intraocular pressure; SD = standard deviation.

**Figure 3:**
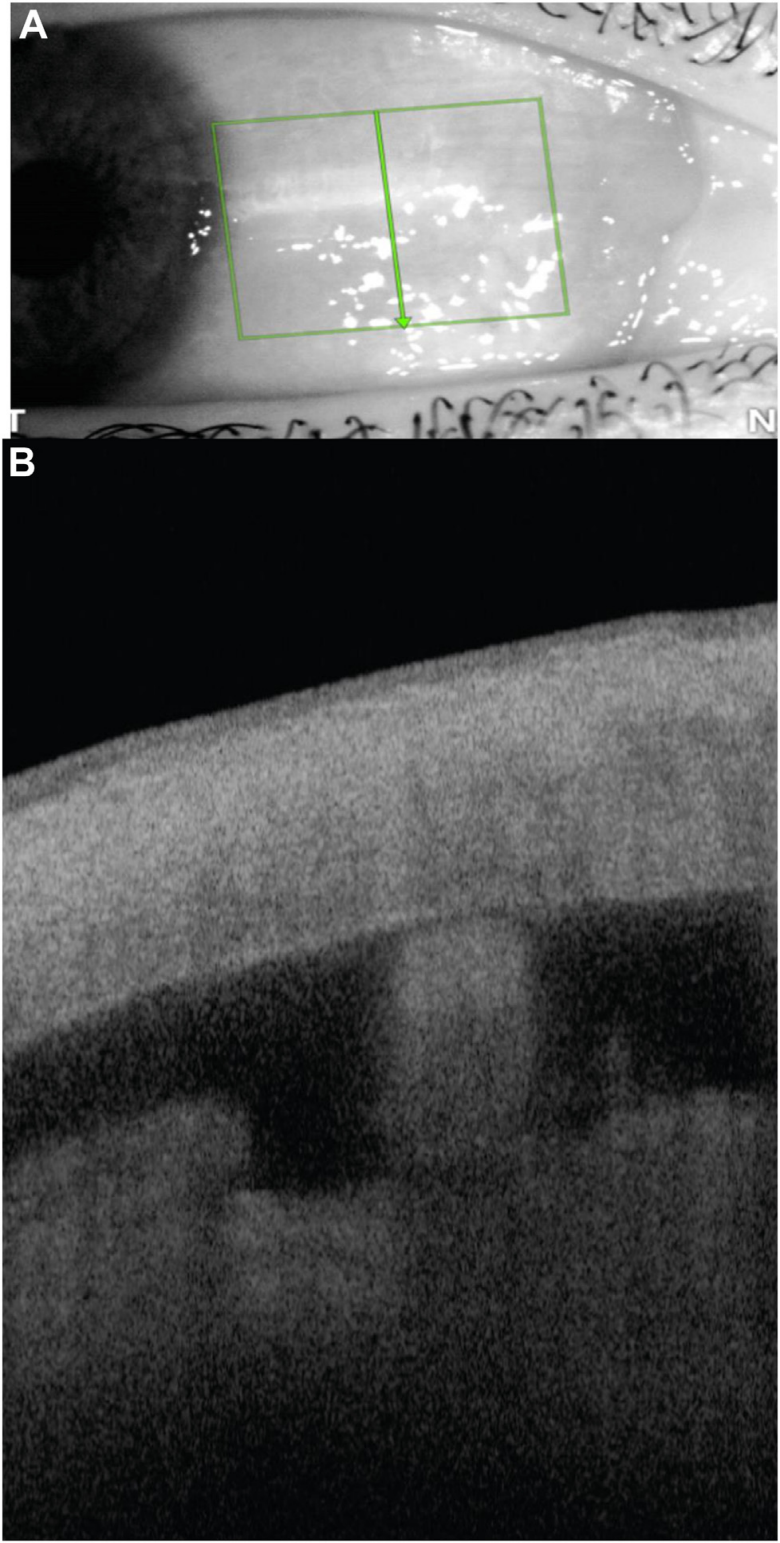
Representative coronal image (green lines and arrow) (A) of scleral reinforcement spacer used to scaffold the cyclodialysis reservoir, with postoperative OCT imaging showing endoscleral bio-integration and tissue homology (B).

All adverse events were transient and occurred in the first 3 to 6 months after surgery (Table 3). There was no clinical hypotony in any of the patients in the early postoperative period and no vision-threatening intraoperative or serious postoperative complications. There was no severe or persistent inflammation or hyphema, and no bio-tissue migration or corneal touch was observed throughout the 24-month follow-up period. Figure 3 illustrates the postoperative OCT imaging, highlighting the stability and tissue compatibility of the bio-stent.

**Table 3:** Adverse Events

Variable	24 Months
Intraoperative hyphema (transient), n (%)	1 (3.2)
Postoperative IOP increase (10 mmHg or >30 mmHg), n (%)∗	2 (6.4)
>2 lines drop in BCVA, n (%)	0
Persistent inflammation requiring topical steroids (>1 mo), n (%)†	5 (16)
Severe postoperative inflammation (grade 4+), n (%)	0
Persistent postoperative hyphema (>1 mo), n (%)	0
Severe postoperative hyphema (>3 mm), n (%)	0
Persistent corneal edema (>1 mo), n (%)	0
Bio-tissue migration, n (%)	0
Cystoid macular edema	0
Hypotony maculopathy	0

BCVA = best-corrected visual acuity; IOP = intraocular pressure.

∗ All resolved with conservative management. One case was on postoperative day 1 secondary to retained viscoelastic, and one case was at 1-month follow-up.

† All cases of mild inflammation beyond the 30-day postoperative period which resolved by 3 months.

## Discussion

This study demonstrates good IOP-lowering effect and long-term durability of the scleral bio-reinforced cyclodialysis combined with cataract surgery. There was a significant and sustained reduction in IOP with few ocular adverse events and complications during both the operative and long-term postoperative periods, aligning with the standards for minimally invasive surgical treatments.

This modified technique addresses key limitations of conventional cyclodialysis by incorporating a bio-reinforcement strategy aimed at improving the consistency and duration of the aqueous outflow. Traditional cyclodialysis, while effective in lowering IOP, has been limited by issues such as fibrosis and failure of the supraciliary filtration conduit.^12^ By contrast, this precisely controlled procedure with the addition of a bio-scaffolding cleft maintainer leads to a more predictable and reliable outcome. The use of advanced microinterventional tools, such as the CycloPen, allows for high precision in disinsertion of the ciliary body over 1 to 2 clock hours. This precise localization, facilitated by high-magnification gonioscopic visualization, minimizes trauma and optimizes the creation of an internal suprachoroidal reservoir, which, when combined with viscocycloplasty, ensures the establishment of a sustainable filtration pathway. Additionally, the incorporation of scleral reinforcement with natural allograft tissue helps to maintain the patency of this reservoir and reduces the risk of fibrotic closure, a common issue with traditional cyclodialysis approaches.

The use of homologous allograft tissue is particularly noteworthy as it offers several advantages over synthetic implants. The biocompatibility and bio-conforming properties of the allograft reduce the likelihood of foreign body reactions, inflammation, and fibrosis.^21^^,^^22^ Furthermore, avoiding the introduction of foreign hardware eliminates the risk of collateral tissue damage, which can be seen with other devices used in MIGS.^26^, ^27^, ^28^ These findings are in line with recent trends in MIGS toward approaches that prioritize safety, biocompatibility, and durability, as reflected in studies that emphasize the long-term advantages of biologically derived materials in ophthalmic surgeries.^19^

The reduction of adverse events during both the perioperative and postoperative periods also supports the safety profile of this technique. With minimal complications, the bio-scaffolded cyclodialysis approach promises a less invasive alternative to more aggressive glaucoma surgeries, such as trabeculectomy or tube shunts, which carry higher risks of postoperative complications.^29^

However, while our findings are promising, they also raise several questions for future investigation. First, a larger cohort will inform further as to the efficacy and safety. Certainly, durability beyond 2 years will need to be established as well, similar to other glaucoma surgeries. Additionally, while uveoscleral outflow has a significant IOP-lowering effect, randomized comparisons with trabecular outflow surgery or incremental to trabecular intervention also are needed. An additional limitation of this prospective single-arm study is its uncontrolled design, which increases the susceptibility of the results to regression-to-the-mean bias. While there may be corneal safety advantages to bio-interventional cyclodialysis without any hardware in the anterior chamber, other differentiating advantages between this approach and conventional suprachoroidal MIGS devices remain to be understood.

The precise mechanisms by which the bio-scaffold prevents fibrotic closure and maintains the long-term patency of the suprachoroidal reservoir require further elucidation. The role of the allograft in modulating the wound-healing response and preventing fibrosis is an area of potential investigation, particularly in understanding how different types of bio-scaffolds influence outcomes. As MIGS evolves, these findings could contribute to the development of even more refined and individualized treatment strategies for patients with glaucoma.

In conclusion, this study demonstrates that the integration of bio-reinforcement into an ab-interno cyclodialysis uveoscleral conduit offers a solution to some limitations associated with traditional cyclodialysis surgery. With sustained IOP reduction, decreased medication reliance, and a favorable safety profile, this approach holds significant potential for broader clinical application. Future studies are needed to validate and define further the treatment profile and clinical utility of this approach.

## References

[1] Heijl A., Leske M.C., Bengtsson B. (2002). Reduction of intraocular pressure and glaucoma progression: results from the Early Manifest Glaucoma Trial. Arch Ophthalmol.

[2] Saheb H., Ahmed I.I. (2012). Micro-invasive glaucoma surgery: current perspectives and future directions. Curr Opin Ophthalmol.

[3] Lim R. (2022). The surgical management of glaucoma: a review. Clin Exp Ophthalmol.

[4] Jayaram H., Kolko M., Friedman D.S., Gazzard G. (2023). Glaucoma: now and beyond. Lancet.

[5] Freeman W. (2023). 2023 glaucoma surgical device market report: global analysis for 2022 to 2028, July, 2023: market scope, glaucoma surgery market: market size, growth forecasts, and market share | market scope. https://www.market-scope.com/pages/reports/393/2023-glaucoma-surgical-device-market-report-global-analysis-for-2022-to-2028-july-2023.

[6] Richter G.M., Coleman A.L. (2016). Minimally invasive glaucoma surgery: current status and future prospects. Clin Ophthalmol.

[7] Birnbaum F.A., Neeson C., Solá-Del Valle D. (2021). Microinvasive glaucoma surgery: an evidence-based review. Semin Ophthalmol.

[8] Lindén C., Alm A. (1999). Prostaglandin analogues in the treatment of glaucoma. Drugs Aging.

[9] Samuelson T.W., Katz L.J., Wells J.M. (2011). Randomized evaluation of the trabecular micro-bypass stent with phacoemulsification in patients with glaucoma and cataract. Ophthalmology.

[10] Nichani P., Popovic M.M., Schlenker M.B. (2021). Microinvasive glaucoma surgery: a review of 3476 eyes. Surv Ophthalmol.

[11] Ahmed I.I.K., Fea A., Au L. (2020). A prospective randomized trial comparing hydrus and iStent microinvasive glaucoma surgery implants for standalone treatment of open-angle glaucoma: the COMPARE study. Ophthalmology.

[12] Weinreb R.N. (2000). Uveoscleral outflow: the other outflow pathway. J Glaucoma.

[13] Böke H. (1990). Zur Geschichte der Zyklodialyse. In memoriam Leopold Heine 1870-1940 [History of cyclodialysis. In memory of Leopold Heine 1870-1940. Klin Monbl Augenheilkd.

[14] O'Brien C.S., Weih J. (1949). Cyclodialysis. Arch Ophthal.

[15] Sewall E.C. (1907). Cyclodialysis for chronic glaucoma. Cal State J Med.

[16] Klemm M., Balazs A., Draeger J., Wiezorrek R. (1995). Experimental use of space-retaining substances with extended duration: functional and morphological results. Graefes Arch Clin Exp Ophthalmol.

[17] Suguro K., Toris C.B., Pederson J.E. (1985). Uveoscleral outflow following cyclodialysis in the monkey eye using a fluorescent tracer. Invest Ophthalmol Vis Sci.

[18] Ianchulev T., Weinreb R.N., Kamthan G. (2024). Biotissue stent for supraciliary outflow in open-angle glaucoma patients: surgical procedure and first clinical results of an aqueous drainage biostent. Br J Ophthalmol.

[19] Denis P., Hirneiß C., Durr G.M. (2022). Two-year outcomes of the MINIject drainage system for uncontrolled glaucoma from the STAR-I first-in-human trial. Br J Ophthalmol.

[20] De Francesco T., Ianchulev T., Rhee D.J. (2024). The evolving surgical paradigm of scleral allograft bio-tissue use in ophthalmic surgery: techniques and clinical indications for ab-externo and ab-interno scleral reinforcement. Clin Ophthalmol.

[21] Chaya C.J., Herndon L.W., Lince J. (2024). Surgical outcomes, ocular safety and tolerability of bio-interventional cyclodialysis with allograft scleral reinforcement: clinical experience of more than 240 cases. J Clin Med.

[22] Watson P.G., Young R.D. (2004). Scleral structure, organisation and disease. A review. Exp Eye Res.

[23] Boote C., Sigal I.A., Grytz R. (2020). Scleral structure and biomechanics. Prog Retin Eye Res.

[24] Olsen T.W., Edelhauser H.F., Lim J.I., Geroski D.H. (1995). Human scleral permeability. Effects of age, cryotherapy, transscleral diode laser, and surgical thinning. Invest Ophthalmol Vis Sci.

[25] Prausnitz M.R., Noonan J.S. (1998). Permeability of cornea, sclera, and conjunctiva: a literature analysis for drug delivery to the eye. J Pharm Sci.

[26] Coudrillier B., Tian J., Alexander S. (2012). Biomechanics of the human posterior sclera: age- and glaucoma-related changes measured using inflation testing. Invest Ophthalmol Vis Sci.

[27] Lass J.H., Benetz B.A., He J. (2019). Corneal endothelial cell loss and morphometric changes 5 Years after phacoemulsification with or without CyPass micro-stent. Am J Ophthalmol.

[28] Ahmed I.I.K., Sheybani A., De Francesco T., Samuelson T.W. (2024). Corneal endothelial safety profile in minimally invasive glaucoma surgery. J Cataract Refract Surg.

[29] Gedde S.J., Feuer W.J., Lim K.S. (2022). Postoperative complications in the primary tube versus trabeculectomy study during 5 Years of follow-up. Ophthalmology.

